# Supplementary material for: Time-Kill Analysis of Canine Skin Pathogens: A Comparison of Pradofloxacin and Marbofloxacin
Source: Antibiotics (Basel). 2023 Oct 17;12(10):1548. doi: 10.3390/antibiotics12101548 (PMC10603860; doi:10.3390/antibiotics12101548)
Supplement: Supplementary file 1 [file antibiotics-12-01548-s001.zip › antibiotics-2647883-supplementary-update.pdf]

**Table S1.** List of isolates used in the study for time-kill analysis. *S. pseudintermedius*, *S. aureus* and *E. coli*, collected from canine skin infection cases.

| Bacterial species | Isolate ID | Methicillin susceptibility[a] | Haemolysis | Marbofloxacin-susceptibility[c] | Marbofloxacin MIC (μg/mL) 2-fold dilution | FQ-resistance genes | | |
|---|---|---|---|---|---|---|---|---|
| | | | | | | *gyrA* | *grlA/parC* | PMQR |
| *S. pseudintermedius* | 22219 | MSSP | Yes | S | 0.25 | WT | WT | NA |
| | 108 | MSSP | Yes | S | 0.5 | WT | WT | NA |
| | 1726 | MRSP | Yes | S | 0.25 | WT | WT | NA |
| | 41 | MRSP | Yes | S | 0.25 | WT | WT | NA |
| | 98 | MSSP | Yes | R | 4 | Glu88Gly | Ser80Arg | NA |
| | 115 | MSSP | Yes | R | 16 | Ser84Leu | Asp84Asn | NA |
| | 38 | MRSP | Yes | R | 32 | Ser84Leu | Ser80Ile | NA |
| | 67 | MRSP | Yes | R | 32 | Ser84Leu | Ser80Ile | NA |
| *S. aureus* | 476 | MSSA | Yes | S | 0.5 | WT | WT | NA |
| | B98 | MSSA | Yes | S | 0.5 | WT | WT | NA |
| | A53 | MRSA | Yes | S | 0.5 | WT | WT | NA |
| | A54 | MRSA | Yes | S | 0.5 | WT | WT | NA |
| | B53 | MSSA | Yes | R | 16 | Ser84Leu | Ser80Phe | NA |
| | B94 | MSSA | Yes | R | 16 | Ser84Leu | Ser80Phe | NA |
| | A009 | MRSA | Yes | R | 32 | Ser84Leu | Ser80Phe | NA |
| | A69 | MRSA | Yes | R | 64 | Ser84Leu, Gly90Cys | Ser80Phe | NA |
| *E. coli* | 14L-1510 | - | Yes | S | 0.06 | WT | WT | NA |
| | 16L-1242 | - | Yes | S | 0.06 | WT | WT | NA |
| | 17L-0826 | - | No | S | 0.03 | WT | WT | NA |
| | 17L-1562 | - | No | S | 0.125 | WT | WT | NA |
| | 2443 | - | No | R | 8 | Ser83Leu | Ser80Ile | None |
| | 10L-2253 | - | No | R | 16 | Ser83Leu, Asp87Asn | Ser80Ile, Ala108Val | None |
| | 10L-3690 | - | No | R | 32 | Ser83Leu, Asp87Asn | Ser80Ile | *aac-(6')-lb-cr* |
| | 15L-3275 | - | No | R | 32 | Ser83Leu, Asp87Asn | Ser80Ile | *qnrB* |

Isolates were chosen based on their susceptibility to marbofloxacin, and those reported as FQ-resistant were screened for the presence of chromosomal mutations on DNA gyrase and Topoisomerase IV, and also for the presence of plasmid-mediated quinolone resistance (PMQR) genes in *E. coli.*

[a] Methicillin resistance was confirmed by the presence of *mecA* gene. [b]WT: wild-type bacteria

[c] susceptibility to marbofloxacin was assessed according to clinical breakpoints from CLSI guidelines VET01S [1]. Resistance was considered if MIC ≥ 4 μg/mL.

**Table S2**. Pharmacokinetic data (free concentration) obtained from preclinical studies in dogs following oral administration of marbofloxacin (2 mg/kg Schneider, *et al.* [2] or pradofloxacin (3 mg/kg Hauschild, *et al.* [3].

| FQ | Dose (mg/kg) | Route | $fC_{max}$ (µg/mL) | $T_{max}$ (hours) | Clearance ($L.kg^{-1}.h^{-1}$) | $AUC_{0-24h}$ ($µg.h.mL^{-1}$) | $AUC_{0-inf}$ ($µg*h.mL^{-1}$) | Protein binding (%) |
|---|---|---|---|---|---|---|---|---|
| Marbofloxacin | 2 | PO | 1.25 | 25 | 0.1 | 14.55 | 19.96 | 9.1 (product monograph)<br>25 (Bregante, Bidgood and Papich) |
| Pradofloxacin | 3 | PO | 1.19 | 2.33 | 0.244 | 10.75 | 12.27 | 36-37 |

$C_{max}$: maximum serum drug concentration. $T_{max}$: time necessary to achieve $C_{max}$. Half-life: time at which the initial drug concentration is reduced by 50%; Clearance: amount of drug that is removed from the body per unit of time; AUC is the area under the concentration curve: Protein binding is the amount of drug bound to plasmatic proteins (data were obtained from separate sources, marbofloxacin (product monographs of Marbocyl® [4] and Zeniquin® [5] and pradofloxacin from Bregante, *et al.* [6].

**Table S3.** Pharmacodynamic parameters in 4 FQ-susceptible and 4 FQ-resistant strains of *S. pseudintermedius* collected from canine pyoderma or skin wound cases.

| *S. pseudintermedius* *Pharmacodynamic parameters* | | | | | | | | | | | |
|---|---|---|---|---|---|---|---|---|---|---|---|
| **PRADOFLOXACIN** | | | | | | **MARBOFLOXACIN** | | | | | |
| | **Estimates** | | **Bootstrap (*n*=30)** | | | | **Estimates** | | **Bootstrap (*n*=30)** | | |
| **$EC_{50}$_P (mg/L)** | **Susceptible Pool** | **Resistant pool** | **Median** | **2.5% CI** | **97.5% CI** | **$EC_{50}$_M (mg/L)** | **Susceptible Pool** | **Resistant pool** | **Median** | **2.5% CI** | **97.5% CI** |
| MSSP_22219 | **0.037** | - | 0.036 | 0.029 | 0.049 | MSSP_22219 | **0.17** | - | 0.18 | 0.15 | 0.21 |
| MSSP_108 | **0.041** | - | 0.041 | 0.032 | 0.054 | MSSP_108 | **0.49** | - | 0.45 | 0.41 | 0.53 |
| MRSP_1726 | **0.033** | - | 0.039 | 0.030 | 0.054 | MRSP_1726 | **0.18** | - | 0.19 | 0.15 | 0.21 |
| MRSP_41 | **0.031** | - | 0.034 | 0.026 | 0.048 | MRSP_41 | **0.18** | - | 0.17 | 0.12 | 0.19 |
| MSSP_98 | - | **0.25** | 0.25 | 0.22 | 0.28 | MSSP_98 | - | **2.63** | 2.79 | 2.58 | 3.06 |
| MSSP_115 | - | **0.82** | 0.82 | 0.69 | 0.90 | MSSP_115 | - | **8.41** | 8.58 | 7.70 | 11.38 |
| MRSP_38 | - | **1.49** | 1.72 | 1.46 | 1.84 | MRSP_38 | - | **19.94** | 22.21 | 19.71 | 32.26 |
| MRSP_67 | - | **1.21** | 1.30 | 1.07 | 1.64 | MRSP_67 | - | **22.83** | 24.00 | 22.02 | 25.42 |
| $E_{max}$_P (1/h) | | | | | | $E_{max}$_M (1/h) | | | | | |
| | **2.23** | - | 2.36 | 1.74 | 2.87 | * | **1.85** | - | 1.91 | 1.53 | 2.04 |
| | - | **1.80** | 1.72 | 1.54 | 2.03 | | - | **1.64** | 1.61 | 1.44 | 1.86 |
| Gamma_P (scalar) | **1.90** | | 1.87 | 1.67 | 2.54 | Gamma_M (scalar) | **2.58** | | 2.58 | 2.30 | 3.01 |
| **Error term** stdev (Ln domain) **1.29** | | | | | | | | | | | |

$E_{max}$, maximal increase in killing rate in addition to $K_{DEATH}$, $EC_{50}$: concentration required to achieve 50% of $E_{max}$; gamma, Hill's coefficient; Emax and gamma differed between susceptible and resistant isolates.

**Table S4.** Pharmacodynamic parameters in 4 FQ-susceptible and 4 FQ-resistant *S. aureus* collected from canine pyoderma or skin wound cases.

| *S. aureus* *Pharmacodynamic parameters* | | | | | | | | | | | |
|---|---|---|---|---|---|---|---|---|---|---|---|
| **PRADOFLOXACIN** | | | | | | **MARBOFLOXACIN** | | | | | |
| | **Estimates** | | **Bootstrap (*n*=30)** | | | | **Estimates** | | **Bootstrap (*n*=30)** | | |
| | **Susceptible Pool** | **Resistant pool** | **Median** | **2.5% CI** | **97.5% CI** | | **Susceptible Pool** | **Resistant pool** | **Median** | **2.5% CI** | **97.5% CI** |
| **$EC_{50}$_P (mg/L)** | | | | | | **$EC_{50}$_M (mg/L)** | | | | | |
| MSSA_476 | **0.061** | - | 0.062 | 0.058 | 0.085 | MSSA_476 | **0.32** | - | 0.317 | 0.297 | 0.359 |
| MSSA_B98 | **0.072** | - | 0.076 | 0.069 | 0.103 | MSSA_B98 | **0.31** | - | 0.31 | 0.29 | 0.36 |
| MRSA_A53 | **0.051** | - | 0.052 | 0.040 | 0.066 | MRSA_A53 | **0.28** | - | 0.28 | 0.26 | 0.32 |
| MRSA_A54 | **0.031** | - | 0.031 | 0.024 | 0.052 | MRSA_A54 | **0.25** | - | 0.25 | 0.22 | 0.28 |
| MSSA_B53 | - | **1.29** | 1.28 | 1.24 | 1.48 | MSSA_B53 | - | **15.47** | 15.74 | 14.78 | 16.85 |
| MSSA_B94 | - | **1.34** | 1.34 | 1.29 | 1.90 | MSSA_B94 | - | **17.05** | 17.77 | 16.15 | 19.16 |
| MRSA_A009 | - | **1.34** | 1.33 | 1.26 | 1.87 | MRSA_A009 | - | **16.30** | 16.72 | 15.53 | 17.65 |
| MRSA_A69 | - | **4.46** | 4.20 | 4.10 | 5.30 | MRSA_A69 | - | **60.60** | 62.30 | 57.22 | 65.31 |
| **$E_{max}$_P (1/h)** | **2.17** | | 2.30 | 1.88 | 2.69 | **$E_{max}$_M (1/h)** | **1.97** | | 2.01 | 1.67 | 2.23 |
| **gamma_P (scalar)** | **2.06** | | 1.98 | 1.14 | 2.68 | **gamma_M (scalar)** | **2.34** | | 2.28 | 1.80 | 2.86 |
| **Error term** | | | | | | | | | | | |
| stdev (Ln domain) **1.53** | | | | | | | | | | | |

$E_{max}$, maximal increase in killing rate in addition to $K_{DEATH}$, $EC_{50}$: concentration required to achieve 50% of $E_{max}$; gamma, Hill's coefficient; Emax and gamma were shared between susceptible and resistant isolates.

**Table S5.** Pharmacodynamic parameters in 4 FQ-susceptible and 4 FQ-resistant *E. coli* collected from canine pyoderma or skin wound cases.
$E_{max}$, maximal increase in killing rate in addition to $K_{DEATH}$, $EC_{50}$_S1: concentration required to achieve 50% of $E_{max}$ for the dominant more susceptible population S1; gamma, Hill's coefficient; coefficient of variation (CV%) and confidence interval (CI) are represented in the Hessian matrix and obtained by simple run model. FOLD is a parameter that represents the potency ratio between the $EC_{50S2}$ and $EC_{50S1}$.

*E. coli Pharmacodynamic parameters*

| Pradofloxacin | Estimates | | Precision of estimates | | |
|---|---|---|---|---|---|
| | Susceptible Pool | Resistant pool | CV% | 2.5% CI | 97.5% CI |
| **$EC_{50}$_P_S1 (mg/L)** | | | | | |
| *E. coli* 14L_1510 | **0.047** | - | 8.72 | 0.04 | 0.05 |
| *E. coli* 16L_1242 | **0.052** | - | 9.13 | 0.04 | 0.06 |
| *E. coli* 17L_0826 | **0.033** | - | 9.43 | 0.03 | 0.04 |
| *E. coli* 17L_1562 | **0.076** | - | 9.29 | 0.06 | 0.09 |
| *E. coli* 2443 | - | **1.81** | 5.62 | 1.61 | 2.01 |
| *E. coli* 10L_2253 | - | **2.07** | 5.88 | 1.83 | 2.30 |
| *E. coli* 10L_3690 | - | **7.43** | 6.69 | 6.45 | 8.41 |
| *E. coli* 15L_3275 | - | **13.38** | 5.37 | 11.97 | 14.80 |
| **FOLD_P** | **1.97** | | | | |
| **$E_{max}$_P (1/h)** | | | | | |
| Susceptible | **8.73** | - | 3.63 | 8.11 | 9.35 |
| Resistant | - | **3.11** | 3.03 | 2.93 | 3.30 |
| **gamma_P (scalar)** | | | | | |
| Susceptible | **1.17** | | 5.58 | 1.04 | 1.30 |
| Resistant | | **2.37** | 10.08 | 1.90 | 2.84 |

| Marbofloxacin | Estimates | | Precision of estimates | | |
|---|---|---|---|---|---|
| | Susceptible pool | Resistant pool | CV% | 2.5% CI | 97.5% CI |
| **$EC_{50}$_M_S1 (mg/L)** | | | | | |
| *E. coli* 14L_1510 | **0.119** | - | 9.01 | 0.10 | 0.14 |
| *E. coli* 16L_1242 | **0.178** | - | 9.80 | 0.14 | 0.21 |
| *E. coli* 17L_0826 | **0.157** | - | 9.74 | 0.13 | 0.19 |
| *E. coli* 17L_1562 | **0.642** | - | 9.88 | 0.52 | 0.77 |
| *E. coli* 2443 | - | **4.03** | 4.40 | 3.69 | 4.38 |
| *E. coli* 10L_2253 | - | **7.81** | 3.50 | 7.27 | 8.34 |
| *E. coli* 10L_3690 | - | **23.55** | 4.55 | 21.45 | 25.66 |
| *E. coli* 15L_3275 | - | **15.91** | 4.43 | 14.53 | 17.29 |
| **FOLD_M** | **1.67** | | | | |
| **$E_{max}$_M (1/h)** | | | | | |
| Susceptible | **17.14** | - | 4.22 | 15.72 | 18.56 |
| Resistant | - | **2.85** | 2.64 | 2.71 | 3.00 |
| **gamma_M (scalar)** | | | | | |
| Susceptible | **1.12** | | 3.91 | 1.03 | 1.20 |
| Resistant | | **2.80** | 8.22 | 2.35 | 3.25 |

**Error term** stdev (Ln domain) **2.17**

**Table S6.** Secondary parameters of 4 FQ-susceptible and 4 FQ-resistant *S. pseudintermedius*.

| *S. pseudintermedius* Secondary parameters | | | | | | | | | | | |
|---|---|---|---|---|---|---|---|---|---|---|---|
| **MIC PRADOFLOXACIN (µg/mL)** | | | | | | **MIC MARBOFLOXACIN (µg/mL)** | | | | | |
| | **Experimental MIC (mg/L)** | **Estimated MIC (mg/L)** | **Median** | **2.5% CI** | **97.5% CI** | | **Experimental MIC (mg/L)** | **Estimated MIC (mg/L)** | **Median** | **2.5% CI** | **97.5% CI** |
| **Susceptible pool** | | | | | | **Susceptible pool** | | | | | |
| MSSP_22219 | 0.025 | **0.037** | 0.036 | 0.027 | 0.039 | MSSP_22219 | 0.40 | **0.20** | 0.20 | 0.19 | 0.25 |
| MSSP_108 | 0.028 | **0.037** | 0.037 | 0.027 | 0.039 | MSSP_108 | 0.35 | **0.52** | 0.51 | 0.41 | 0.54 |
| MRSP_1726 | 0.031 | **0.030** | 0.032 | 0.027 | 0.041 | MRSP_1726 | 0.30 | **0.19** | 0.19 | 0.18 | 0.23 |
| MRSP_41 | 0.025 | **0.028** | 0.028 | 0.027 | 0.034 | MRSP_41 | 0.30 | **0.19** | 0.18 | 0.15 | 0.20 |
| **Resistant pool** | | | | | | **Resistant pool** | | | | | |
| MSSP_98 | 0.225 | **0.28** | 0.28 | 0.24 | 0.32 | MSSP_98 | 2.80 | **3.15** | 3.18 | 3.03 | 3.95 |
| MSSP_115 | 0.9 | **0.93** | 0.89 | 0.84 | 0.97 | MSSP_115 | 11.20 | **10.07** | 10.29 | 9.28 | 13.25 |
| MRSP_38 | 1.8 | **1.70** | 1.80 | 1.55 | 2.39 | MRSP_38 | 32.00 | **23.86** | 25.77 | 22.36 | 40.53 |
| MRSP_67 | 1.6 | **1.37** | 1.39 | 1.26 | 2.00 | MRSP_67 | 25.6 | **27.31** | 27.95 | 25.95 | 31.30 |

Minimum inhibitory concentrations were estimated by the model and compared with experimentally measured MICs. Median and confidence intervals were obtained through bootstrap analysis.

**Table S7.** Secondary parameters of 4 FQ-susceptible and 4 FQ-resistant *S. aureus*.

| *S. aureus* Secondary parameters | | | | | | | | | | | |
|---|---|---|---|---|---|---|---|---|---|---|---|
| PRADOFLOXACIN | | | | | | MARBOFLOXACIN | | | | | |
| | Experimental MIC (mg/L) | Estimated MIC (µg/mL) | Median | 2.5% CI | 97.5% CI | | Measured MIC (µg/mL) | Estimated MIC (µg/mL) | Median | 2.5% CI | 97.5% CI |
| **Susceptible pool** | | | | | | **Susceptible pool** | | | | | |
| MSSA_476 | 0.056 | **0.059** | 0.059 | 0.051 | 0.066 | MSSA_476 | 0.4 | **0.34** | 0.33 | 0.30 | 0.37 |
| MSSA_B98 | 0.05 | **0.064** | 0.064 | 0.055 | 0.075 | MSSA_B98 | 0.35 | **0.30** | 0.30 | 0.27 | 0.32 |
| MRSA_A53 | 0.031 | **0.045** | 0.043 | 0.035 | 0.048 | MRSA_A53 | 0.3 | **0.28** | 0.27 | 0.24 | 0.29 |
| MRSA_A54 | 0.028 | **0.028** | 0.027 | 0.022 | 0.030 | MRSA_A54 | 0.3 | **0.24** | 0.24 | 0.21 | 0.26 |
| **Resistant pool** | | | | | | **Resistant pool** | | | | | |
| MSSA_B53 | 1.8 | **1.15** | 1.136 | 0.715 | 1.195 | MSSA_B53 | 12.8 | **15.11** | 15.00 | 13.56 | 16.92 |
| MSSA_B94 | 1.4 | **1.19** | 1.191 | 0.899 | 1.308 | MSSA_B94 | 14.4 | **16.65** | 16.89 | 15.15 | 18.00 |
| MRSA_A009 | 1 | **1.19** | 1.172 | 0.928 | 1.236 | MRSA_A009 | 19.2 | **15.92** | 15.90 | 13.93 | 17.22 |
| MRSA_A69 | 3.6 | **3.96** | 3.834 | 2.349 | 4.072 | MRSA_A69 | 51.2 | **59.17** | 58.62 | 50.02 | 64.83 |

Minimum inhibitory concentrations were estimated by the model and compared with experimentally measured MICs. Median and confidence intervals were obtained through bootstrap analysis.

**Table S8.** Secondary parameters of 4 FQ-susceptible and 4 FQ-resistant *E. coli*.

| | **MIC pradofloxacin** | | | | | | | **MIC marbofloxacin** | | | | | |
|---|---|---|---|---|---|---|---|---|---|---|---|---|---|
| | **Experimental MIC (µg/mL)** | **Dominant susceptible population** | **Subdominant less susceptible population** | **CV%** | **2.5% CI** | **97.5% CI** | | **Experimental MIC (µg/mL)** | **Dominant susceptible population** | **Subdominant less susceptible population** | **CV%** | **2.5% CI** | **97.5% CI** |
| **susceptible pool** | | | | | | | **susceptible pool** | | | | | | |
| *E. coli* 14L_1510 | 0.022 | **0.013** | **0.026** | 5.46 | 0.012 | 0.015 | *E. coli* 14L_1510 | 0.025 | **0.016** | **0.026** | 4.96 | 0.014 | 0.017 |
| *E. coli* 16L_1242 | 0.022 | **0.014** | **0.029** | 5.54 | 0.013 | 0.016 | *E. coli* 16L_1242 | 0.025 | **0.023** | **0.039** | 5.24 | 0.021 | 0.026 |
| *E. coli* 17L_0826 | 0.013 | **0.009** | **0.018** | 5.79 | 0.008 | 0.010 | *E. coli* 17L_0826 | 0.025 | **0.021** | **0.034** | 5.30 | 0.018 | 0.023 |
| *E. coli* 17L_1562 | 0.030 | **0.021** | **0.042** | 5.80 | 0.019 | 0.024 | *E. coli* 17L_1562 | 0.100 | **0.084** | **0.140** | 5.41 | 0.075 | 0.093 |
| **Resistant pool** | | | | | | | **Resistant pool** | | | | | | |
| *E. coli* 2443 | 2.40 | **1.85** | **3.94** | 5.47 | 1.65 | 2.05 | *E. coli* 2443 | 5.60 | **4.40** | **7.35** | 4.78 | 3.99 | 4.82 |
| *E. coli* 10L_2253 | 2.80 | **2.12** | **4.18** | 5.70 | 1.88 | 2.35 | *E. coli* 10L_2253 | 14.40 | **8.52** | **14.21** | 3.83 | 7.88 | 9.16 |
| *E. coli* 10L_3690 | 9.60 | **7.61** | **15.02** | 6.43 | 6.65 | 8.57 | *E. coli* 10L_3690 | 32.00 | **25.71** | **42.88** | 4.91 | 23.23 | 28.18 |
| *E. coli* 15L_3275 | 19.20 | **13.71** | **27.06** | 5.24 | 12.30 | 15.12 | *E. coli* 15L_3275 | 22.40 | **17.36** | **28.96** | 4.80 | 15.73 | 19.00 |

Minimum inhibitory concentrations were estimated by the model and compared with experimentally measured MICs. Coefficient of variation (CV%) and confidence interval (CI) are represented in the Hessian matrix and obtained by simple run model.

**Table S9.** Critical PK/PD values ($fAUC_{PK\_0\text{-}24h}$/MIC) that achieve 50% and 90% of the maximal antibacterial effect from the *in silico* model.
Two representative isolates (susceptible and resistant) were chosen for each bacterial species (*S. pseudintermedius, S. aureus and E. coli*). Concentration is a unitless value which represents the average free plasma concentration required over 24 h to achieve 90% of the maximal efficacy.

# Dose fractionation

| **Isolate** | **PRADOFLOXACIN** | | | | **MARBOFLOXACIN** | | | |
|---|---|---|---|---|---|---|---|---|
| | **Experimental MIC (µg/mL)** | **$fAUC_{PK\_0\text{-}24h}$/MIC (h)** | | **Average concentration (µg/mL) to achieve 90% of $I_{max}$** | **Experimental MIC (µg/mL)** | **$fAUC_{PK\_0\text{-}24h}$/MIC (h)** | | **Average concentration (µg/mL) to achieve 90% of $I_{max}$** |
| | | **50%** | **90%** | | | **50%** | **90%** | |
| **MSRP 41 (susceptible)** | 0.025 | 24.97 | 35.72 | 0.037 | 0.18 | 25.75 | 35.91 | 0.26 / **0.26** |
| **MRSP 67 (resistant)** | 1.60 | 19.44 | 29.53 | 1.97 | 25.60 | 25.23 | 36.19 | 39.78 / **38.76** |
| **MSSA B98 (susceptible)** | 0.050 | 31.41 | 44.95 | 0.094 | 0.35 | 21.30 | 31.65 | 0.92 / **0.46** |
| **MSSA B53 (resistant)** | 1.80 | 15.75 | 22.86 | 1.71 | 12.80 | 29.51 | 43.83 | 23.38 / **22.83** |
| ***E. coli* 14L-1510 (susceptible)** | 0.022 | 17.04 | 26.36 | 0.024 | 0.025 | 17.11 | 26.91 | 0.024 / **0.017** |
| ***E. coli* 10L-2253 (resistant)** | 2.80 | 24.11 | 31.45 | 3.67 | 14.40 | 17.21 | 23.11 | 13.87 / **13.89** |

Footnote: *S. pseudintermedius*: $fAUC_{PK\_0\text{-}24h}$/MIC was the best PK/PD index for both susceptible ($R^2$ 0.987, AIC 52.61 for pradofloxacin and $R^2$ 0.990, AIC 44.94 for marbofloxacin) and resistant isolates ($R^2$ 0.992, AIC 44.80 pradofloxacin and $R^2$ 0.990, AIC 48.02 for marbofloxacin) for in both FQs.
*S. aureus:* $fAUC_{PK\_0\text{-}24h}$/MIC was the best PK/PD index for both susceptible ($R^2$ 0.987, AIC 52.61 pradofloxacin and $R^2$ 0.990, AIC 44.94 for marbofloxacin) and resistant isolates ($R^2$ 0.992, AIC 44.80 pradofloxacin and $R^2$ 0.990, AIC 48.02 for marbofloxacin) for marbofloxacin in both FQs.
*E. coli:* $fAUC_{PK\_0\text{-}24h}$/MIC was the best PK/PD index for both susceptible ($R^2$ 0.996, AIC 23.08 pradofloxacin and $R^2$ 0.990, AIC 66.52 for marbofloxacin) and resistant isolates ($R^2$ 0.996, AIC 23.29 pradofloxacin and $R^2$ 0.989, AIC 59.15).

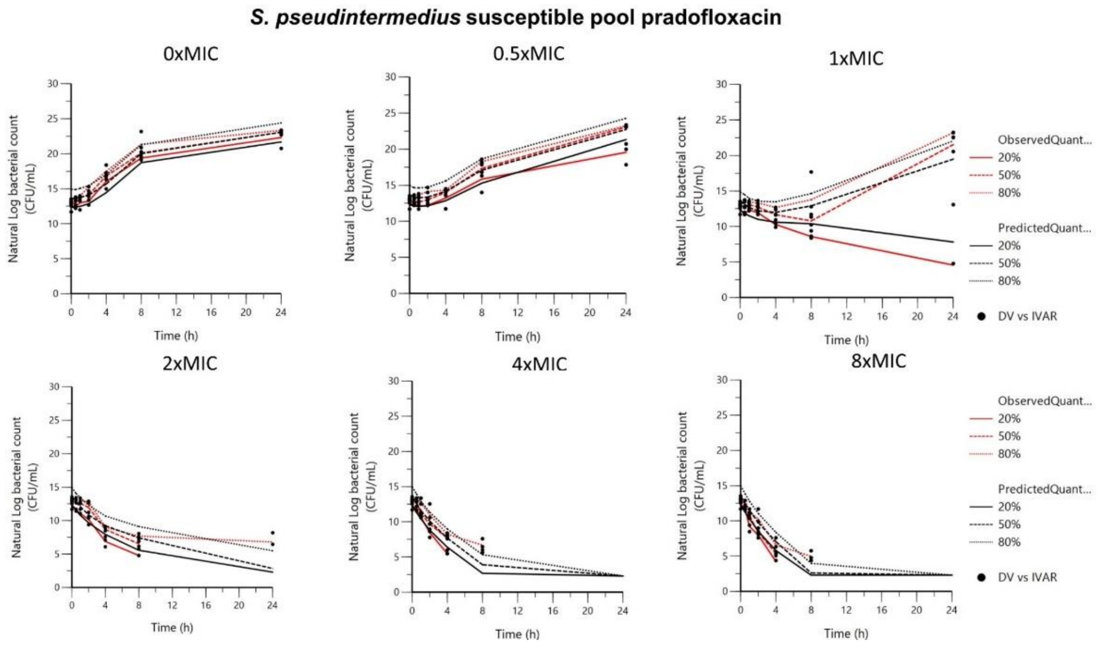


**Figure S1** Visual predictive check (VPC) of pradofloxacin-susceptible *S. pseudintermedius.* Each stratification for each observed quantiles (20, 50 and 80%, red lines) are superimposed with the predicted quantiles (20,50 and 80%, black lines). Black dots represent the observed data.

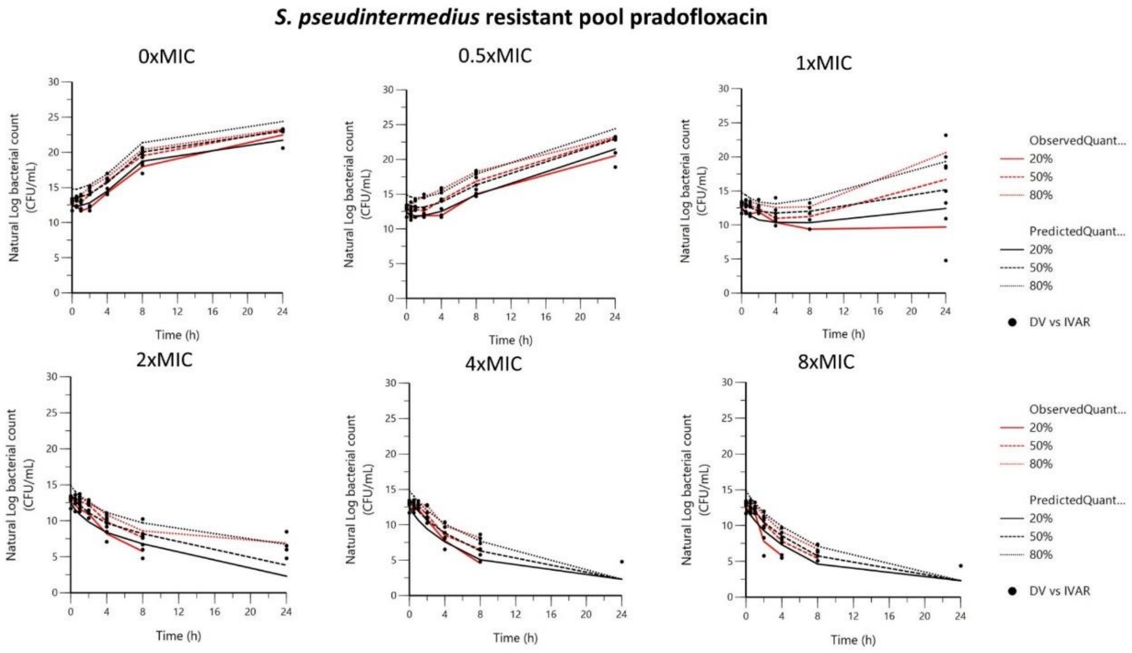


**Figure S2** Visual predictive check (VPC) of pradofloxacin-susceptible *S. pseudintermedius*. Each stratification for each observed quantiles (20, 50 and 80%, red lines) are superimposed with the predicted quantiles (20,50 and 80%, black lines). Black dots represent the observed data.

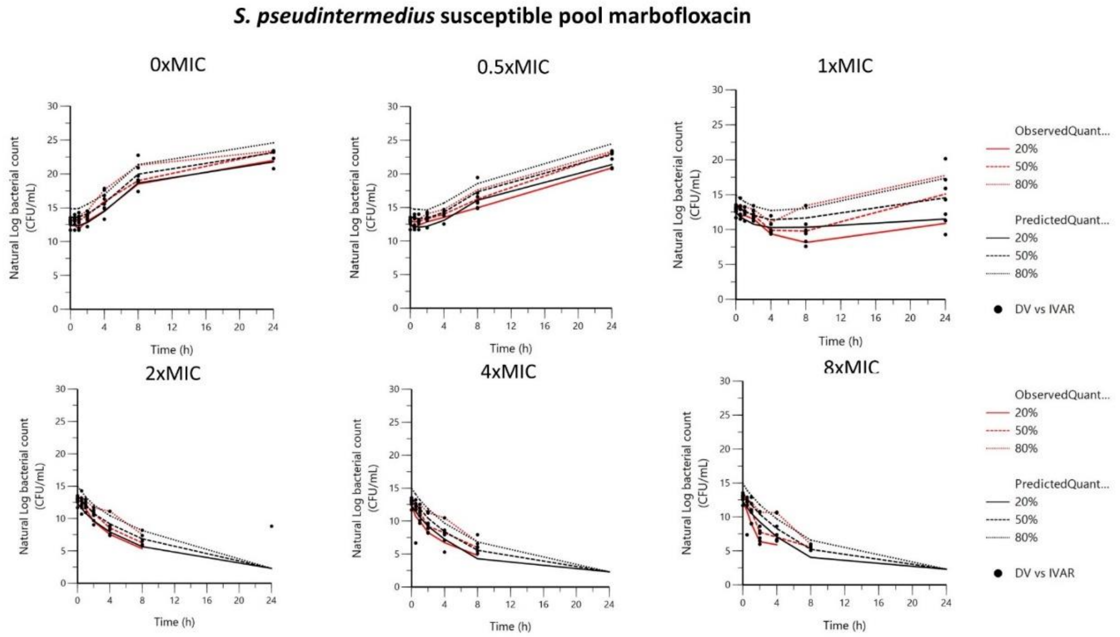


**Figure S3.** Visual predictive check (VPC) of pradofloxacin-resistant *S. pseudintermedius*.
Each stratification for each observed quantiles (20, 50 and 80%, red lines) are superimposed with the predicted quantiles (20,50 and 80%, black lines). Black dots represent the observed data.

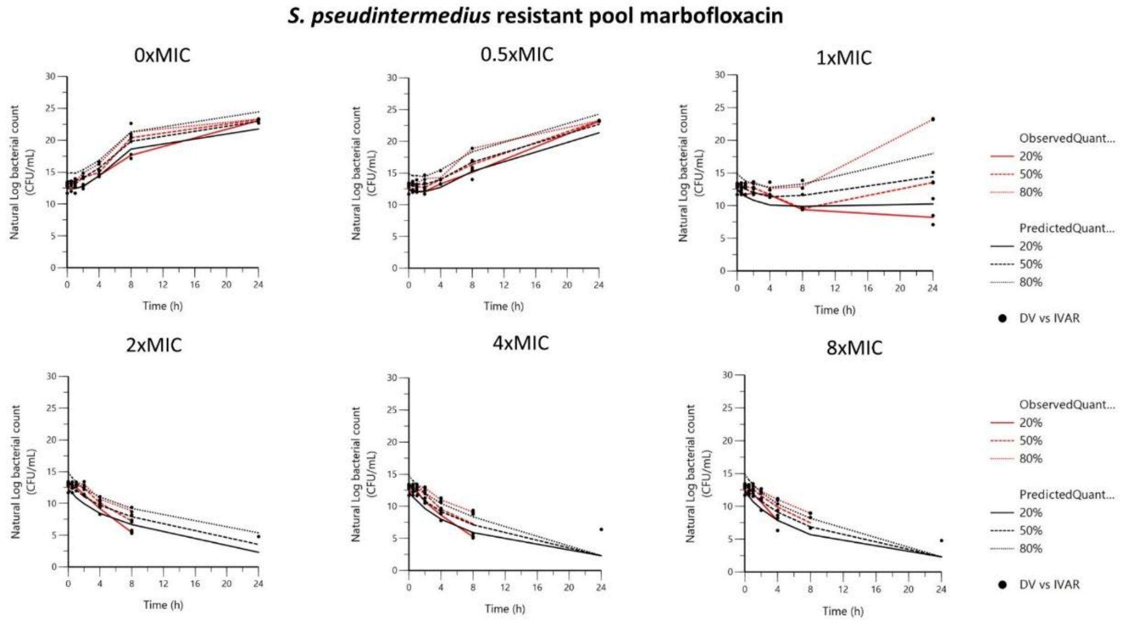


**Figure S4.** Visual predictive check (VPC) of marbofloxacin-resistant *S. pseudintermedius*.
Each stratification for each observed quantiles (20, 50 and 80%, red lines) are superimposed with the predicted quantiles (20,50 and 80%, black lines). Black dots represent the observed data.

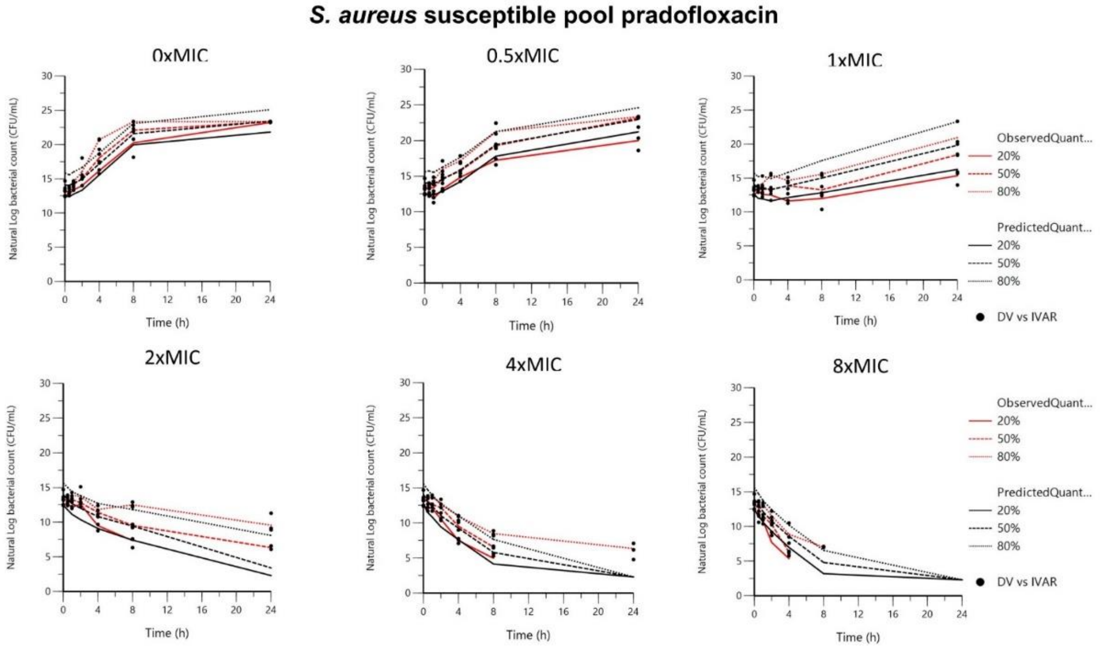


**Figure S5.** Visual predictive check (VPC) of pradofloxacin-susceptible *S. aureus.*
Each stratification for each observed quantiles (20, 50 and 80%, red lines) are superimposed with the predicted quantiles (20,50 and 80%, black lines). Black dots represent the observed data.

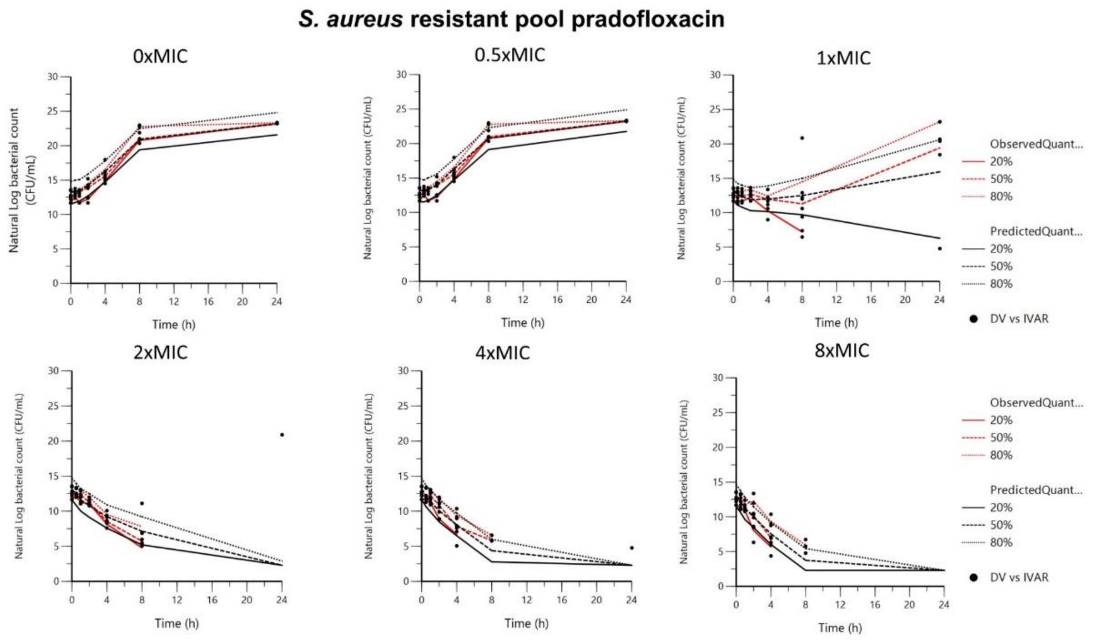


**Figure S6.** Visual predictive check (VPC) of pradofloxacin-resistant *S. aureus.*
Each stratification for each observed quantiles (20, 50 and 80%, red lines) are superimposed with the predicted quantiles (20,50 and 80%, black lines). Black dots represent the observed data.

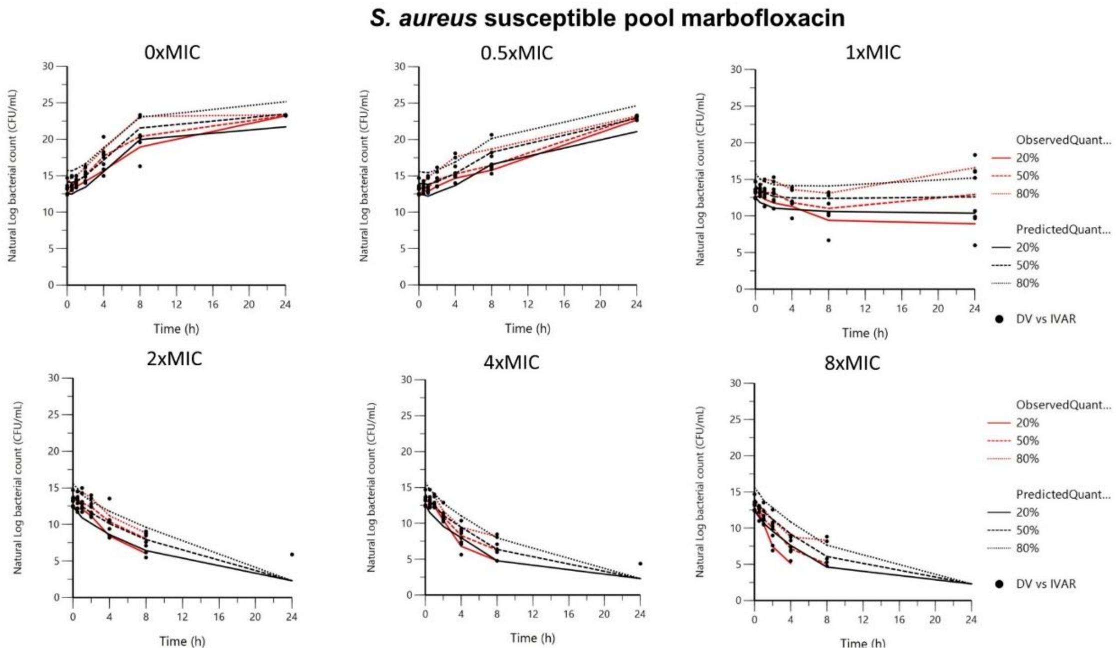


**Figure S7.** Visual predictive check (VPC) of marbofloxacin-susceptible *S. aureus.*
Each stratification for each observed quantiles (20, 50 and 80%, red lines) are superimposed with the predicted quantiles (20,50 and 80%, black lines). Black dots represent the observed data.

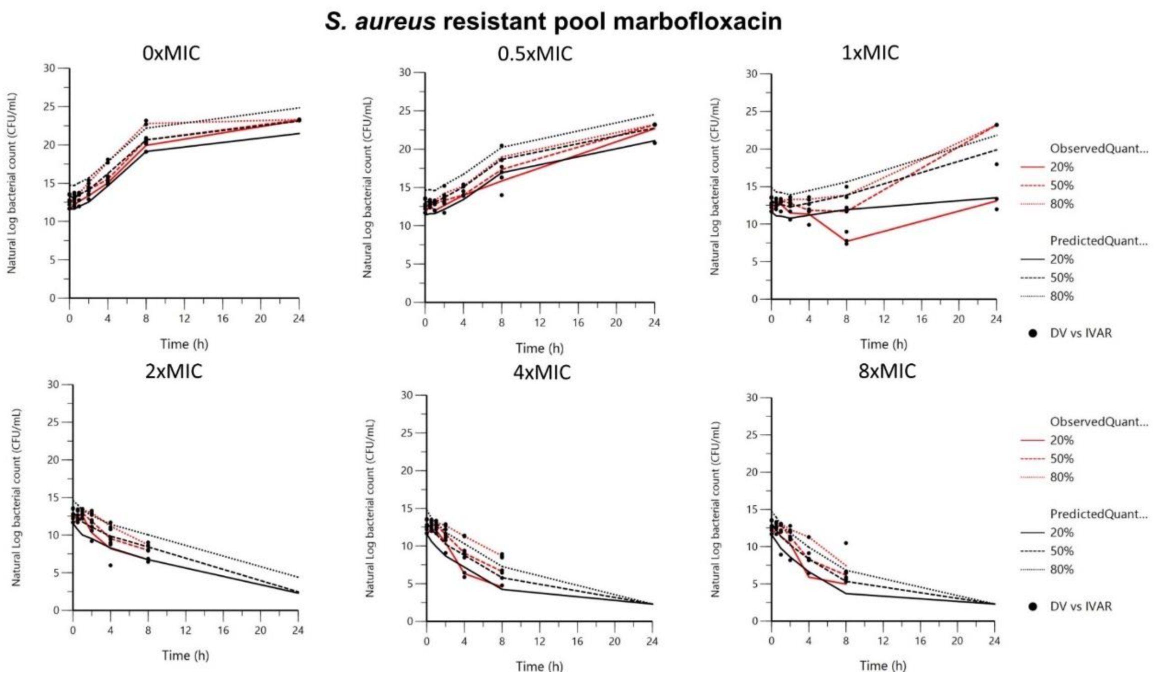


**Figure S8.** Visual predictive check (VPC) of marbofloxacin resistant *S. aureus.*
Each stratification for each observed quantiles (20, 50 and 80%, red lines) are superimposed with the predicted quantiles (20,50 and 80%, black lines). Black dots represent the observed data.

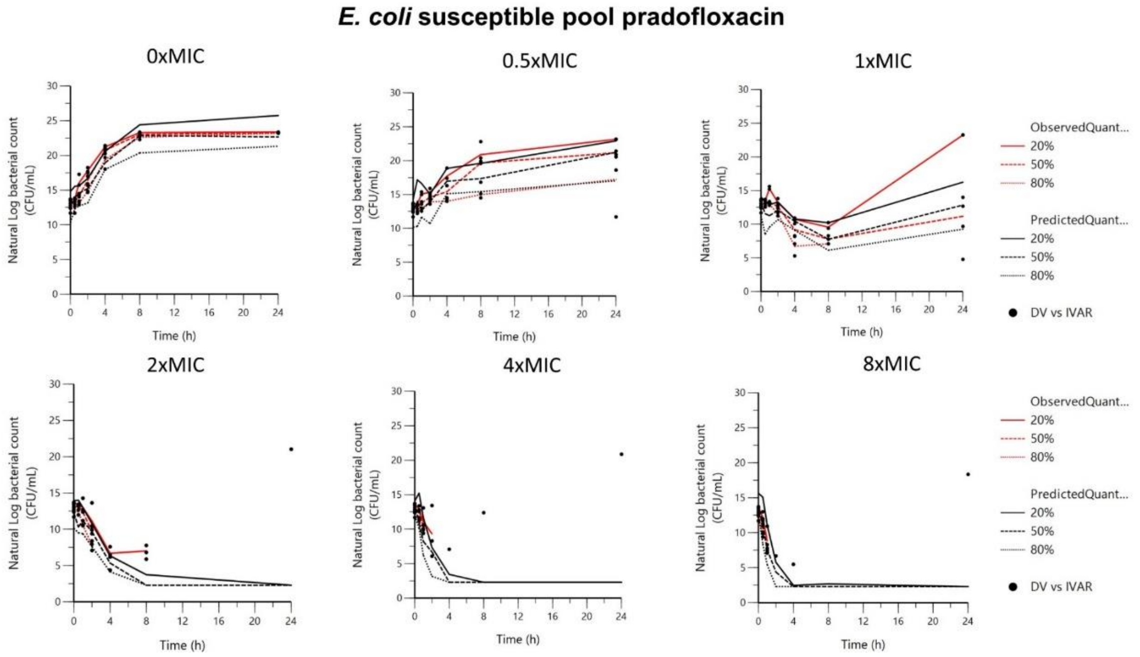


**Figure S9.** Visual predictive check (VPC) of pradofloxacin-susceptible *E. coli.*
Each stratification for each observed quantiles (20, 50 and 80%, red lines) are superimposed with the predicted quantiles (20,50 and 80%, black lines). Black dots represent the observed data.

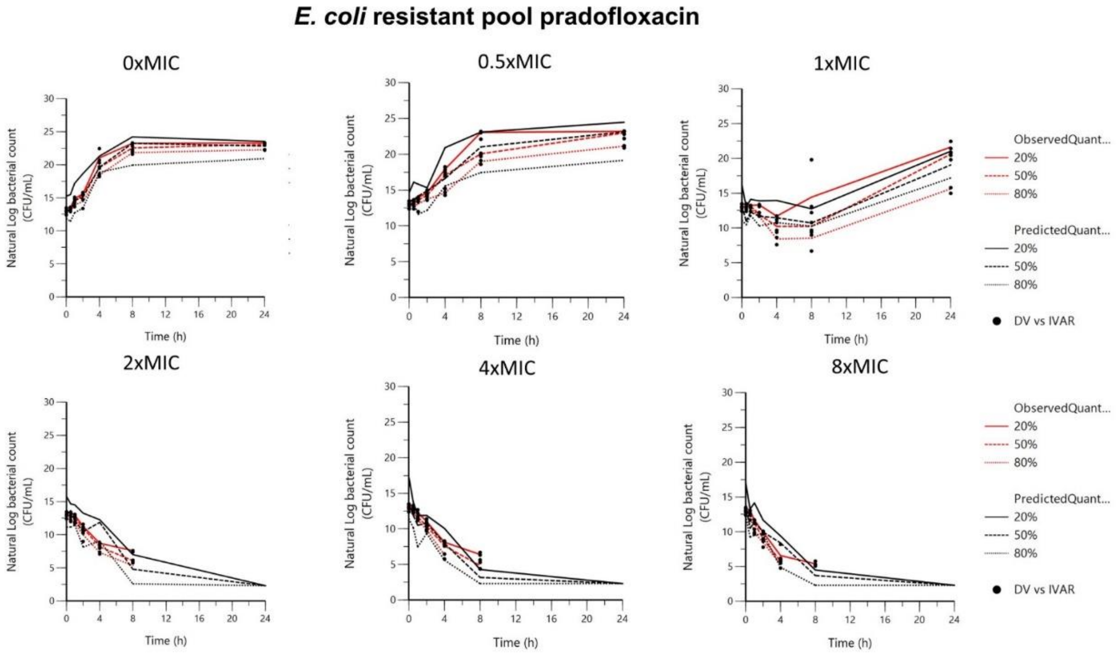


**Figure S10.** Visual predictive check (VPC) of pradofloxacin-resistant *E. coli.*
Each stratification for each observed quantiles (20, 50 and 80%, red lines) are superimposed with the predicted quantiles (20,50 and 80%, black lines). Black dots represent the observed data.

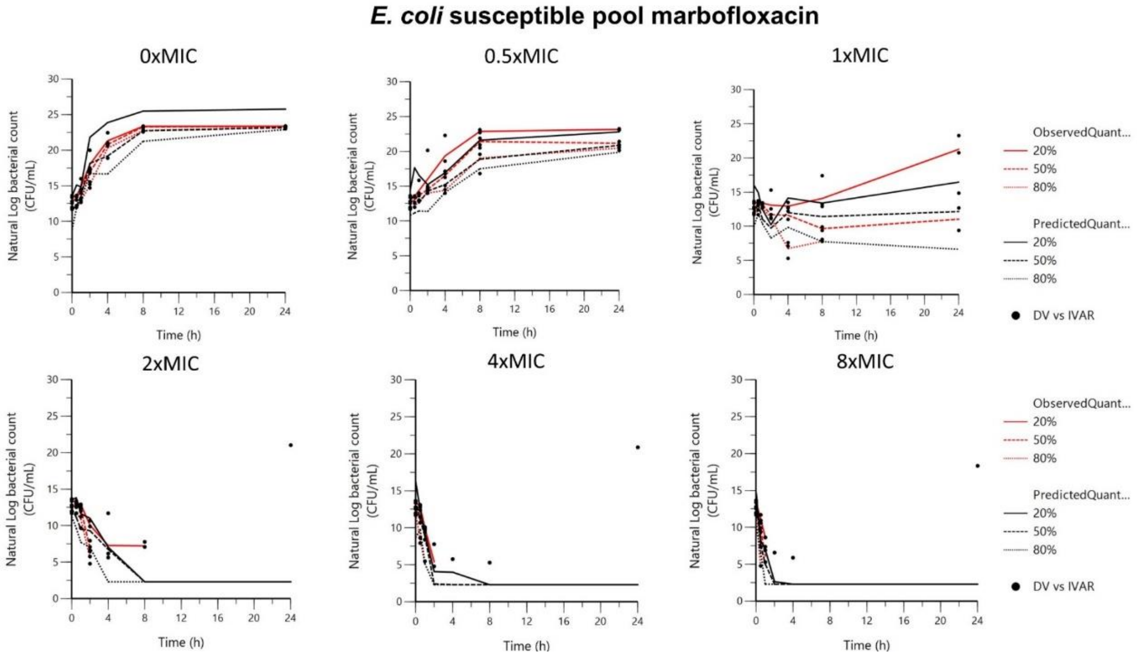


**Figure S11.** Visual predictive check (VPC) of marbofloxacin-susceptible *E. coli*.
Each stratification for each observed quantiles (20, 50 and 80%, red lines) are superimposed with the predicted quantiles (20,50 and 80%, black lines). Black dots represent the observed data.

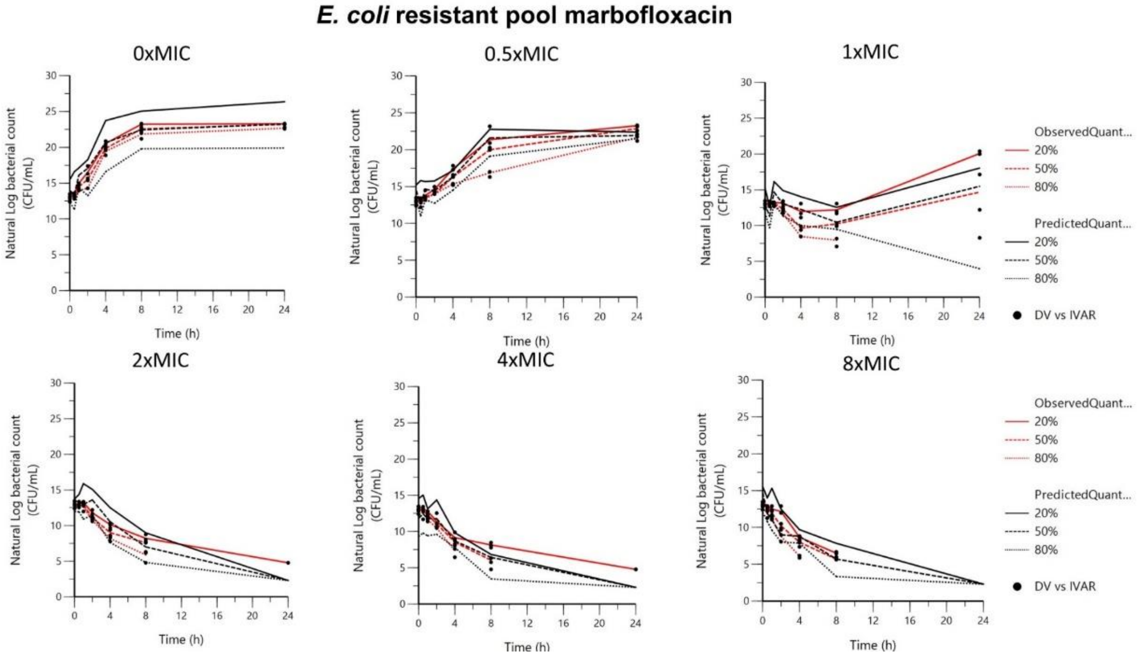


**Figure S12.** Visual predictive check (VPC) of marbofloxacin-resistant *E. coli*.
Each stratification for each observed quantiles (20, 50 and 80%, red lines) are superimposed with the predicted quantiles (20,50 and 80%, black lines). Black dots represent the observed data.

**Figure S13.** Predicted bacterial count (IPRED, expressed) versus observed bacterial count (DV) in 4 FQ-susceptible and 4 FQ-resistant *S. pseudintermedius*. Data are expressed as CFU/mL and are log normal transformed. Observed vs predicted values (black dots) should ideally be aligned to the unity line (y=x)

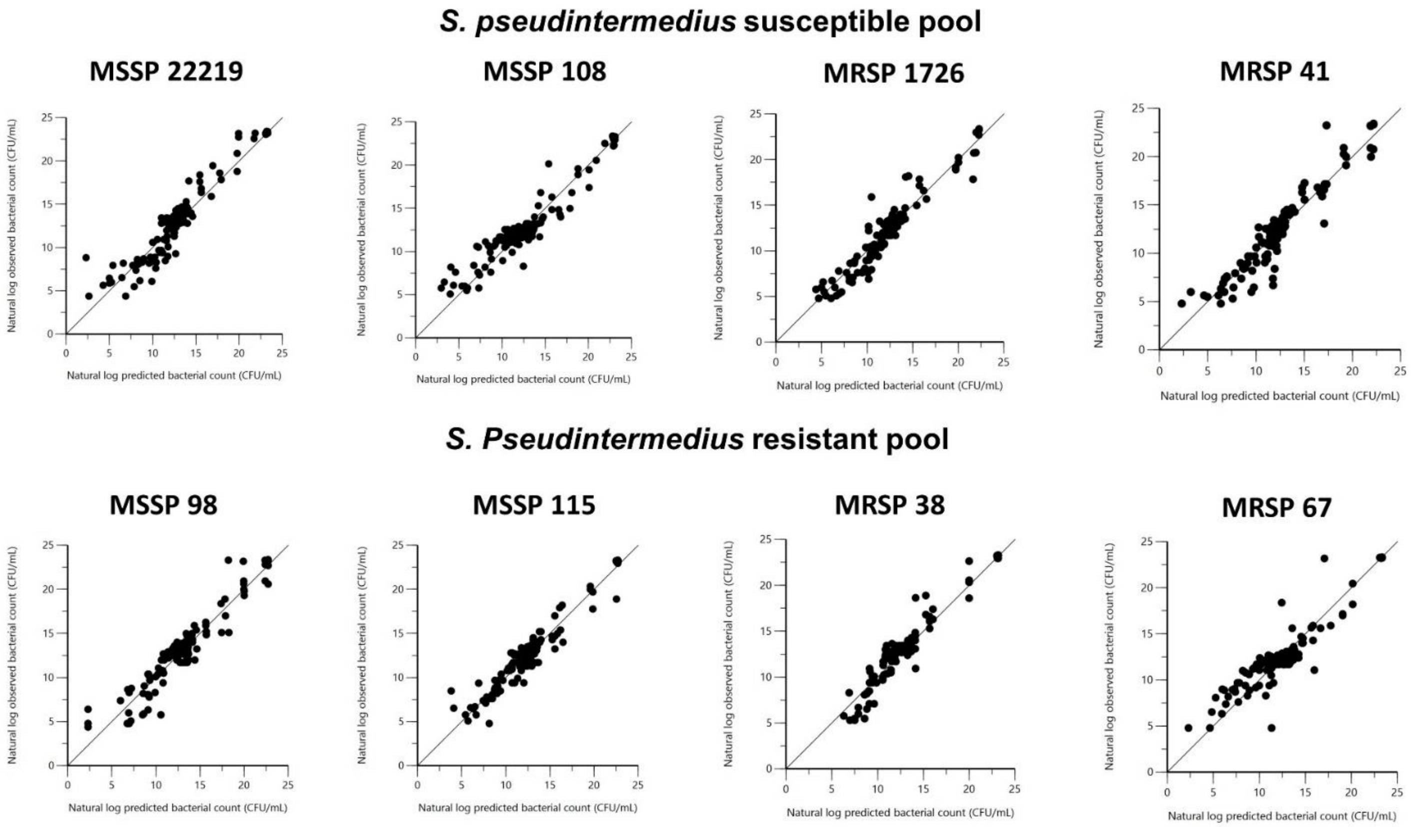

**Figure S14.** Predicted bacterial count (IPRED, expressed) versus observed bacterial count (DV) in 4 FQ-susceptible and 4 FQ-resistant *S. aureus*. Data are expressed as CFU/mL and are log normal transformed. Observed vs predicted values (black dots) should ideally be aligned to the unity line (y=x).

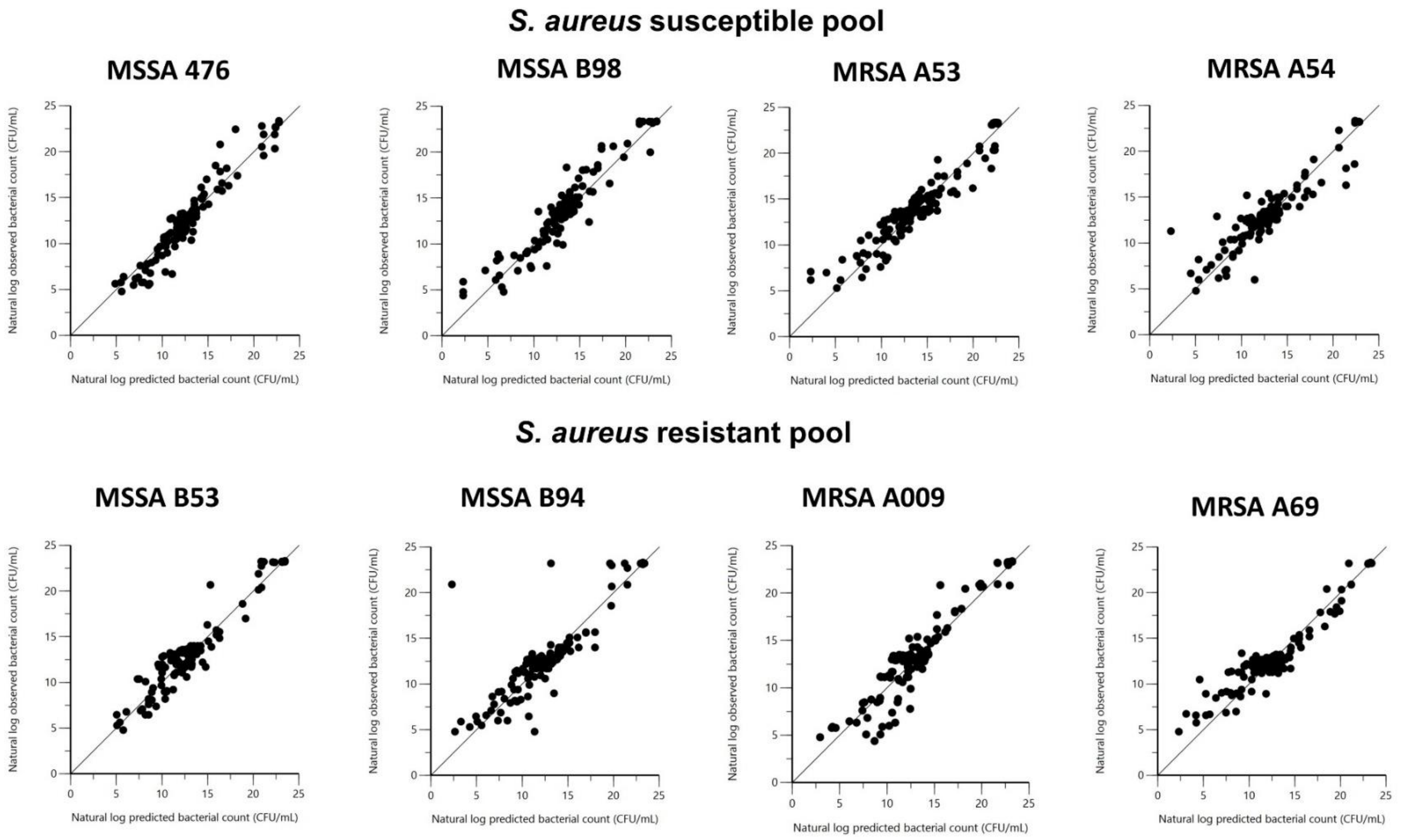

**Figure S15.** Predicted bacterial count (IPRED, expressed) versus observed bacterial count (DV) in 4 FQ-susceptible and 4 FQ-resistant *E. coli*. Data are expressed as CFU/mL and are log normal transformed. Observed vs predicted values (black dots) should ideally be aligned to the unity line (y=x).

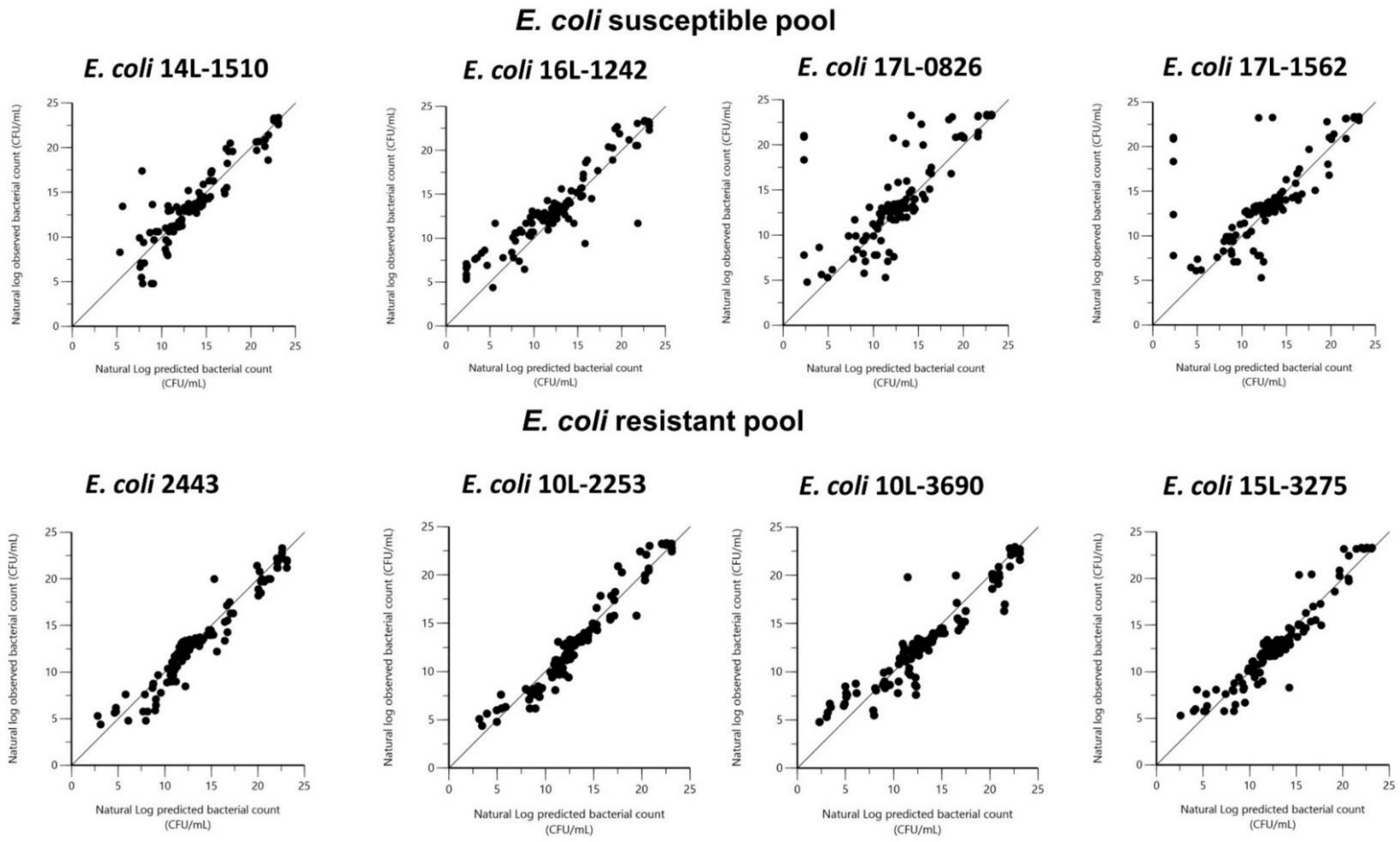

**Figure S16.** Plot of CWRES (conditional weighted residuals) and IWRES (individual weighted residuals) vs Predicted bacterial count (PRED) for the three bacterial species used in the study. CWRES/IWRES represent the goodness of statistical fit and values should be concentrated between y= –2 and y=+2. Values that are above 3 or below –3 may indicate a lack of fit.

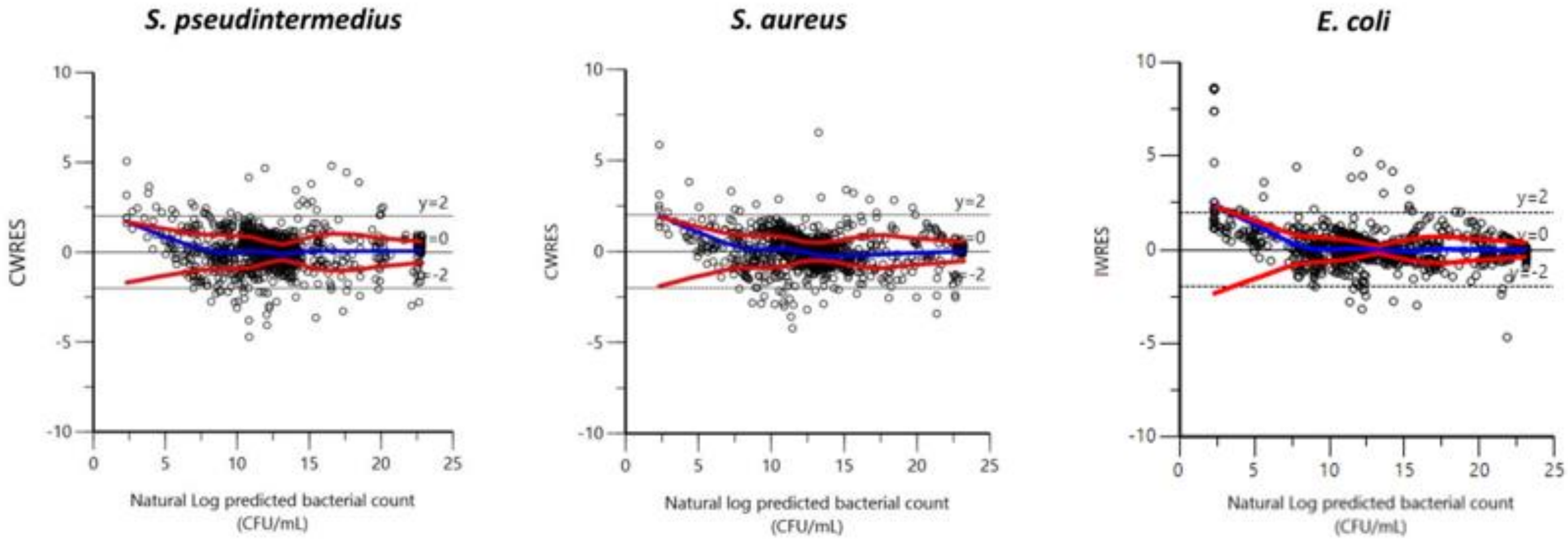

**Figure S17.** Simple linear regression between experimental (x axis) and model-estimated MICs in *S. pseudintermedius*, *S. aureus* and *E. coli* collected from canine skin infection cases.

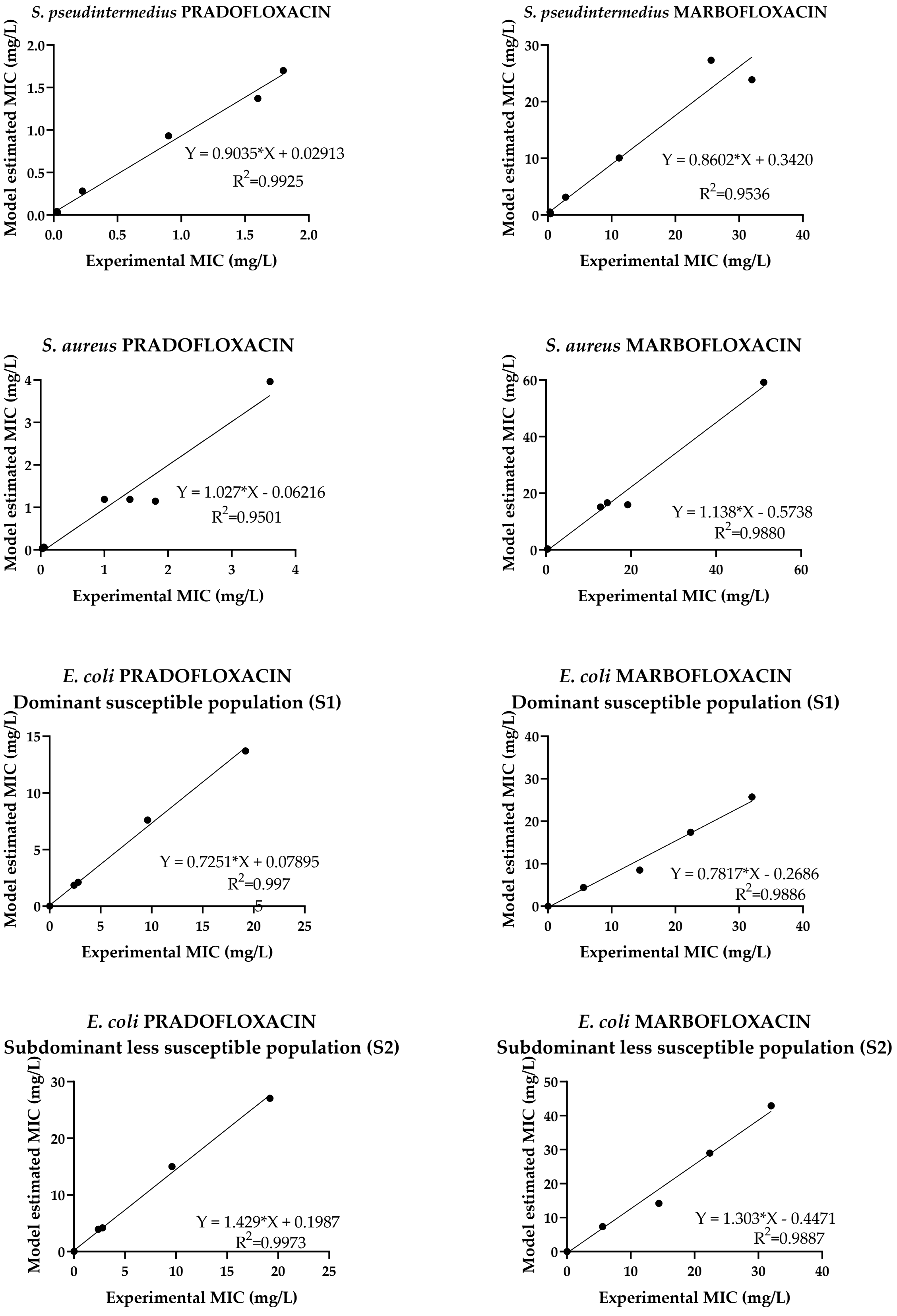

**Text S1 Mathematical modelling**

*Bacterial growth model*

Compartment "S" has a growth rate constant, ($k_{growth}$, expressed in $h^{-1}$). To capture the progressive increase of $K_{growth}$ from 0 to a maximal value ($k_{growthmax}$), $k_{growth}$ was adjusted based on the following equation:

$$K_{growth} = K_{growthmax} \text{ x } (1 - e^{-(alpha \times time)})$$

Where alpha, expressed in $h^{-1}$, represents the progressive increase in $k_{growth}$ that reaches a maximum rate, expressed as $k_{growthmax}$. The corresponding mean generation corresponds to ln2 / $k_{growth}$ (expressed in h).

The maximal bacterial carrying capacity of the system (populations S and P) is referred to as $B_{max}$ (observed plateau in bacterial density). During bacterial growth, the semi-mechanistic model predicts that a proportion of the bacteria from the susceptible compartment are transferred to the P compartment at a constant rate called $k_{SP}$. No growth is assumed in the P compartment.

Moreover, both compartments share the same death rate constant ($k_{death}$, expressed in $h^{-1}$). To maintain good identifiability of model parameters, the value of this parameter has traditionally been fixed to 0.179 $h^{-1}$ by previous authors [7-9] and we allocated this value to $k_{death}$ in our models.

*Drug effect model*

The model also included drug effect, considered as additional to $K_{death}$. It is expressed as $k_{drug}$ (unit $h^{-1}$) through the Hill's equation:

$$K_{drug} = \frac{(E_{max} \times C^{\gamma})}{({EC_{50}}^{\gamma} + C^{\gamma})}$$

where, $E_{max}$ is the maximum killing rate effect ($h^{-1}$), $EC_{50}$ (mg/L) is the concentration of the drug able to reach 50% of $E_{max}$ and gamma (scalar), the Hill's coefficient. These parameters represent efficacy, potency and sensitivity of the tested antibiotics, respectively. To account for the differences in measured MIC (related to drug potency), we estimated a separate value of $EC_{50}$ for each isolate.

*Mathematical modelling*

The following differential equations describe the change in number of viable bacteria over time in the S and P compartments:

$$\frac{dS}{dt} = k_{growth} \text{ x S} - (K_{death} + K_{drug}) \text{ x S} - K_{SP} \text{ x S}$$

$$\frac{dP}{dt} = K_{SP} \text{ x S} - K_{death} \text{ x P}$$

The constant of transfer between S and P is described by the following equation:

$$K_{SP} = \frac{(k_{growth} - K_{death})}{B_{max}} \times (S + P)$$

All data obtained in a TKC were simultaneously analysed with a Non-linear Mixed Effect Model (NLMEM).
As differences in $B_{max}$ were observed between isolates for the staphylococci (only), a random component was included in the model with a Between Isolate Variability (BIV), which was modelled using an exponential model of the form:

$$\theta_{Bmax\_i} = \theta_{tv\_Bmax} \times \text{Exp}(\eta_i)$$

With $\theta_i$ is the value of theta (here $B_{max}$) in the $i^{th}$ isolate, $\theta_{tv\_Bmax}$ is the typical population value of this theta and $\eta_i$, the deviation (noted eta) associated to the $i^{th}$ isolate from the corresponding theta population value.

An exponential error model was selected to model residual variability to account for the wide range of possible bacterial counts according to the following equation:

$$Y_{ij} = \hat{Y}_{ij} \text{ x } e^{\varepsilon ij}$$

$\hat{Y}_{ij}$ represents the $j^{th}$ response (expressed in CFU/mL), which is calculated in the $i^{th}$ curve with no initial log transformation of raw data, whereas $\varepsilon_{ij}$ represents the common errors terms with a mean of 0 and a variance of $\sigma^2{}_1$. If an exponential model is specified, and if there is only one error model as for our analysis, the predictions and observations were automatically log-transformed by the software and are fit in that space. This is because the error model becomes additive in log-space, which allows for higher performance and accuracy. This affects all the plots results and residuals because they are in log-space.
Precision of parameter estimates was estimated by computing standard error (SE) using the Hessian method. When the engine was unable to return these values, precision of parameters and their confidence intervals were estimated by a bootstrap method (*n* = 30 samples).

*Model fitting and diagnostic plots*

Candidate models were assessed by goodness-of-fit plots (PRED (population prediction) and IPRED (individual prediction) versus dependent variable (i.e. time), and by inspection of residuals CWRES (conditional weighted residuals).
Moreover, Visual Predictive Check (VPC) allowed graphical comparisons of the $20^{th}$, $50^{th}$ (median) and $80^{th}$ percentiles between model-predicted intervals and observed data, VPC were derived from *n*=200 simulated data sets, stratified by drug and initial bacterial pool (marbofloxacin-susceptible or resistant isolate).

*Secondary parameters*

MIC and MBC were estimated through the following equations, introduced by Mouton and Vink [10]:

$$MIC = EC_{50} \times \left(\frac{K_{growth} - 0.221}{E_{max} - (K_{growth} - 0.221)}\right)^{\frac{1}{\gamma}}$$

The constant 0.221 was obtained from the following equation:

$$\frac{1}{Time\ of\ measurement(24h)} \times LN\left(\frac{N(t)}{N(0)}\right) = 0.221$$

Where N(t) represented the inoculum size at time 18h, which was set at $10^8$ CFU/mL (bacterial density associated with visible growth) and N(0) the initial inoculum of $5x10^5$ CFU/mL.

**Pre-existing heterogenous population model**

The initial model proposed by Nielsen and Friberg [11] did not capture the regrowth of bacteria observed at 1XMIC. We therefore hypothesised that pre-existing subdominant less susceptible bacteria were present within the total population *E. coli.*, as described by Campion et al. [12]. The ratio between subdominant less susceptible bacteria and total bacteria population has been shown from growth curves to be approximately $10^{-8}$ to $10^{-9}$ [13]. The initial susceptible population (starting inoculum) consisted of two subpopulations representing a heterogenous bacterial population with a proportion (F1) of bacteria being a highly susceptible dominant population (S1) and the remaining sub-dominant population (S2) having a lower susceptibility. F1 was estimated by the model. Apportioning of the starting inoculum (SLoad) to each of the initial sub-populations is defined by the following equations:

Proportion of initial inoculum that is apportioned to subpopulation S1.

$$\mathrm{S1} = \mathrm{SLoad} \times \mathrm{F_1}$$

Proportion of initial inoculum that is apportioned to subpopulation S2.

$$\mathrm{S2} = \mathrm{SLoad} \times (1 - \mathrm{F_1})$$

A proportion of the susceptible population (S1 and S2) is irreversibly transferred to a non-susceptible, non-growing population at a rate constant ($K_{SP}$). Both S1 and S2 subpopulations were assumed to have a similar growth constant ($K_{growth}$) and natural death rate ($K_{death}$). Drug effect $K_{drugS1}$ and $K_{drugS2}$ were described by two separate $E_{max}$ sigmoid models, including respectively $E_{maxS1}$, $\gamma_{S1}$ and $EC_{50S1}$ and $E_{maxS2}$, $\gamma_{S2}$ and $EC_{50S2}$. The potency ratio between $EC_{50S2}$ and $EC_{50S1}$ was estimated by the parameter "FOLD", whose drug-specific values were shared between all isolates.

$$EC_{50\_S2} = FOLD \times EC_{50\_S1}$$

## References


1. CLSI-VET01S. Performance Standards for Antimicrobial Disk and Dilution Susceptibility Tests for Bacteria Isolated From Animals, 5th Edition - CLSI VET01S. **2020**.
2. Schneider, M.; Thomas, V.; Boisrame, B.; Deleforge, J. Pharmacokinetics of marbofloxacin in dogs after oral and parenteral administration. *J. Vet. Pharmacol. Ther.* **1996**, *19*, 56-61.
3. Hauschild, G.; Rohn, K.; Engelhardt, E.; Sager, M.; Hardes, J.; Gosheger, G. Pharmacokinetic study on pradofloxacin in the dog–Comparison of serum analysis, ultrafiltration and tissue sampling after oral administration. *BMC Vet. Res.* **2013**, *9*, 32.
4. Marbocyl®. Product monograph https://www.noahcompendium.co.uk/?id=-459540.
5. Zeniquin®. Product monograph: https://www2.zoetisus.com/content/_assets/docs/vmips/package-inserts/zeniquin.pdf (accessed on 01/07/2023).
6. Bregante, M.A.; De Jong, A.; Calvo, A.; Hernandez, E.; Rey, R.; Garcia, M.A. Protein binding of pradofloxacin, a novel 8-cyanofluoroquinolone, in dog and cat plasma. *J. Vet. Pharmacol. Ther.* **2003**, *26*, 87-88.
7. Nielsen, E.I.; Cars, O.; Friberg, L.E. Pharmacokinetic/pharmacodynamic (PK/PD) indices of antibiotics predicted by a semimechanistic PKPD model: a step toward model-based dose optimization. *Antimicrobial agents and chemotherapy* **2011**, *55*, 4619-4630.
8. Nielsen, E.I.; Viberg, A.; Löwdin, E.; Cars, O.; Karlsson, M.O.; Sandström, M. Semimechanistic pharmacokinetic/pharmacodynamic model for assessment of activity of antibacterial agents from time-kill curve experiments. *Antimicrobial agents and chemotherapy* **2007**, *51*, 128-136.
9. Khan, D.D.; Lagerbäck, P.; Cao, S.; Lustig, U.; Nielsen, E.I.; Cars, O.; Hughes, D.; Andersson, D.I.; Friberg, L.E. A mechanism-based pharmacokinetic/pharmacodynamic model allows prediction of antibiotic killing from MIC values for WT and mutants. *J. Antimicrob. Chemother.* **2015**, *70*, 3051-3060.
10. Mouton, J.W.; Vinks, A.A. Relationship between minimum inhibitory concentration and stationary concentration revisited: growth rates and minimum bactericidal concentrations. *Clinical pharmacokinetics* **2005**, *44*, 767-769.
11. Nielsen, E.I.; Friberg, L.E. Pharmacokinetic-pharmacodynamic modeling of antibacterial drugs. *Pharmacol. Rev.* **2013**, *65*, 1053-1090.
12. Campion, J.J.; McNamara, P.J.; Evans, M.E. Pharmacodynamic modeling of ciprofloxacin resistance in Staphylococcus aureus. *Antimicrobial agents and chemotherapy* **2005**, *49*, 209-219.
13. Singh, R.; Ledesma, K.R.; Chang, K.-T.; Hou, J.-G.; Prince, R.A.; Tam, V.H. Pharmacodynamics of moxifloxacin against a high inoculum of Escherichia coli in an in vitro infection model. *Journal of antimicrobial chemotherapy* **2009**, *64*, 556-562.
